# Supplementary material for: The interactome of intact mitochondria by cross-linking mass spectrometry provides evidence for coexisting respiratory supercomplexes
Source: Mol Cell Proteomics. 2017 Dec 8;17(2):216–32. doi: 10.1074/mcp.RA117.000470 (PMC5795388; doi:10.1074/mcp.RA117.000470)
Supplement: Supplemental Data [file supp_RA117.000470_133922_0_supp_23978_fzffwf.zip › spectra_annotation/mito_DR_spectra_annotation/390-1-3-1-3-1.pdf]

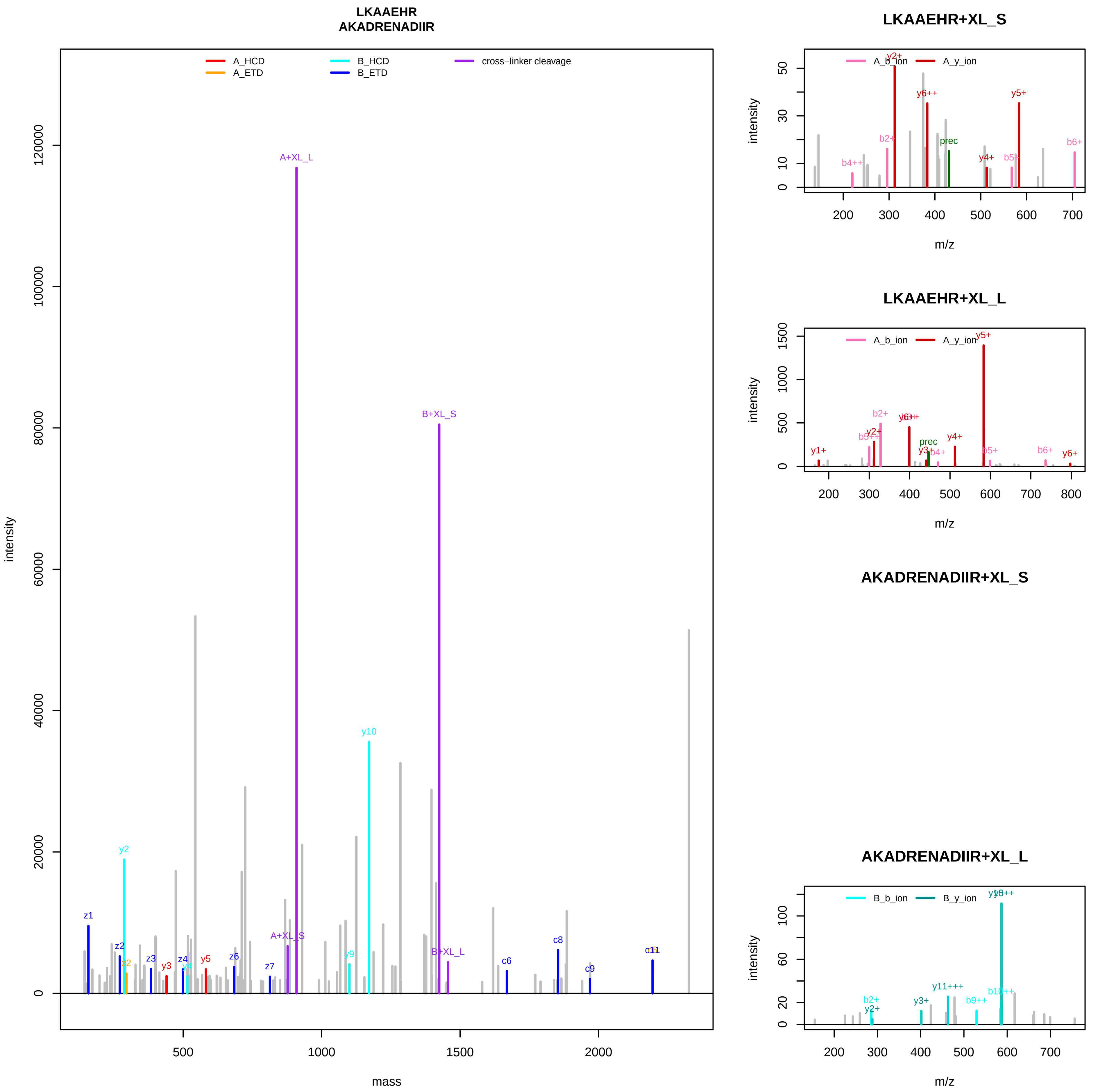
LKAAEHR
AKADRENADIIR
A_HCD
A_ETD
B_HCD
B_ETD
cross−linker cleavage
A+XL_L
B+XL_S
A+XL_S
B+XL_L
y10
y2
z1
z2
z3
z4
z6
z7
y3
y5
y9
c6
c8
c9
c11
intensity
mass
LKAAEHR+XL_S
A_b_ion
A_y_ion
y2+
y6++
y5+
b2+
b4++
prec
y4+
b5+
b6+
m/z
LKAAEHR+XL_L
y1+
b2+
y6++
y2+
b3++
y3+
b4+
y4+
b5+
b6+
y6+
AKADRENADIIR+XL_S
AKADRENADIIR+XL_L
B_b_ion
B_y_ion
b2+
y2+
y3+
y11+++
b9++
b10++
